# Seasonal variation of anti-*Leishmania infantum* antibodies and laboratory abnormalities in dogs with leishmaniosis

**DOI:** 10.1186/s13071-025-06940-7

**Published:** 2025-08-01

**Authors:** Maria Alfonsa Cavalera, Annamaria Uva, Mariaelisa Carbonara, Jairo Alfonso Mendoza-Roldan, Xavier Roura, José Joaquín Cerón, Domenico Otranto, Andrea Zatelli

**Affiliations:** 1 Department of Veterinary Medicine, University of Bari, Valenzano, Italy; 2 Hospital Clínic Veterinari, Universitat Autònoma de Barcelona, Bellaterra, Spain; 3 Laboratory of Clinical Analysis (Interlab-UMU), Regional Campus of International Excellence “Campus Mare Nostrum”, University of Murcia, Murcia, Spain; 4 Department of Veterinary Clinical Sciences, City University of Hong Kong, Hong Kong, SAR China

**Keywords:** APP, Canine leishmaniosis, Clinicopathological alteration, Transmission period, Vector

## Abstract

**Background:**

In dogs affected by leishmaniosis, laboratory abnormalities and anti-*Leishmania* antibody titers are crucial for initial and relapse diagnosis, as well as for informing therapeutic decisions. This study aimed to evaluate laboratory findings in *L. infantum* seropositive dogs during and after the transmission season on the basis of the evidence that anti-*L. infantum* antibody titers vary between sand fly and nonsand fly transmission periods in dogs from areas where leishmaniosis is endemic.

**Methods:**

In September 2021 (transmission season; T1) and January 2022 (nontransmission season; T2), *L. infantum* seropositive dogs were physically examined, and blood sampling was performed for laboratory tests. At both time points, dogs underwent routine hematology, a complete biochemical panel including acute phase proteins (i.e., C-reactive protein [CRP] and ferritin), erythrocyte sedimentation rate [ESR] measurement, serum capillary electrophoresis, and serology for *L. infantum*. Potential coinfections with other arthropod-borne (*Anaplasma phagocythophilum*, *Ehrlichia canis*, *Dirofilaria* spp.) and snail-borne (*Angiostrongylus vasorum*) pathogens were also excluded.

**Results:**

Total protein and CRP levels were slightly reduced in T2 compared with T1, although the difference was not statistically significant. Antibody titers also decreased in 10 out of 18 dogs (55.5%), with two (20%) becoming seronegative, while they remained constant in eight out of 18 dogs (44.4%). Furthermore, a statistically significant reduction was observed in globulin percentage, ferritin, and ESR, whereas albumin percentage and total iron levels significantly increased.

**Conclusions:**

Anti-*L. infantum* antibody titers and laboratory abnormalities in seropositive dogs living in endemic areas for leishmaniosis may vary. This variation may be related to vector seasonality and, consequently, dogs’ exposure to sand fly saliva and potential reinfections. These results reinforce the importance of considering the sampling season in the clinical evaluation and management of dogs affected by leishmaniosis to avoid misdiagnosis and unnecessary antileishmanial treatments.

**Graphical Abstract:**

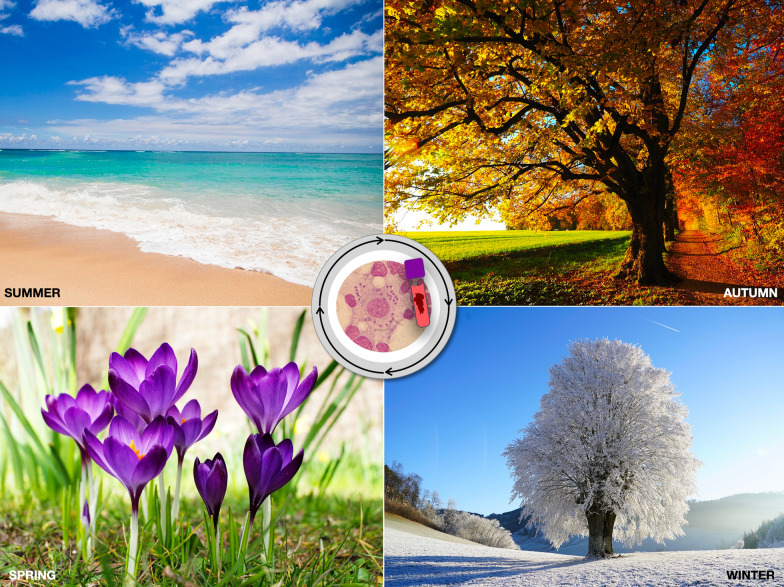

## Background

Canine leishmaniosis (CanL), caused by *Leishmania infantum*, is a multisystemic vector-borne disease endemic in the Mediterranean Basin, South America, Central, and Southwest Asia, where phlebotomine sand fly vectors perpetuate [1]. This chronic infection is characterized by a peculiar clinical polymorphism, ranging from no or mild clinical signs to multiorgan involvement and renal failure [2]. The wide spectrum of CanL outcomes depends mainly on the individual immune response of the affected dog, with disease progression in “susceptible” dogs related to both marked humoral and impaired cellular immune responses against *L. infantum* infection [3].

Clinical suspicion of CanL relies on clinical signs and/or laboratory abnormalities compatible with the disease [4–6]. Confirmation of the infection can be obtained using a combination of direct and/or indirect laboratory diagnostic methods, such as cytology, histopathology, polymerase chain reaction (PCR), and serology [4]. In this regard, according to questionnaire-based surveys completed by veterinary practitioners, quantitative serological techniques such as the indirect immunofluorescence antibody test (IFAT), enzyme-linked immunosorbent assay (ELISA), and/or immunochromatography tests are considered the initial diagnostic approach in general daily practice [7–9]. However, along with serology, laboratory tests such as routine hematology, clinical chemistry, serum protein electrophoresis, and urinalysis are used systematically by veterinary practitioners for both initial and relapse diagnosis of CanL [7, 9].

Typical CanL-related laboratory findings uncovered by these tests include, among others, mild-to-moderate nonregenerative anemia, hyperproteinemia, hyperglobulinemia (i.e., polyclonal beta and/or gammaglobulinemia), hypoalbuminemia, decreased albumin/globulin ratio, renal azotemia, elevated liver enzyme activities, and mild-to-severe proteinuria [4–6]. As part of innate immunity, modifications in acute-phase protein (APP) concentration have also been observed in CanL, being characterized by increases in C-reactive protein (CRP), serum ferritin, and haptoglobin, and decreases in albumin, paraoxonase 1, and apolipoprotein A1 [4, 10, 11]. Moreover, dogs affected by active leishmaniosis present higher erythrocyte sedimentation rate (ESR) levels compared with those exposed or healthy [12]. The increased ESR level is likely associated with an acute phase response, thus a marked increase in APP (e.g., CRP and serum ferritin) circulating in the blood [12].

Furthermore, laboratory alterations are used in conjunction with clinical manifestations, cytology/histopathology, specific serology, and/or PCR for CanL clinical staging, therapeutic monitoring, and prognosis forecasting in dogs with leishmaniosis [2, 6]. For instance, even in the absence of detectable physical signs, a dog should be considered “sick” when presenting with hematologic, biochemical, or urinary alterations suggestive of leishmaniosis [4, 13]. In the latter case, animals require treatment with an appropriate antileishmanial protocol [13]. Hence, the results of the laboratory findings and serology, along with their interpretation, play a pivotal role in the clinical management of CanL [4, 6, 13]. For example, anti-*L. infantum* antibody titers decreased in a population of dogs living in an endemic area, from sand fly to nonsand fly transmission periods, with half of them becoming seronegative [14]. A similar pattern was recorded in domestic ferrets in Spain [15], eventually concluding that such a decrease in anti-*L. infantum* antibody titers may be related to the progressive reduction of exposure to vectors [14]. More in particular, the immune response of the host could be likely upregulated during the transmission period owing to uninfected and *L. infantum*-infected sand fly bites, and the immunogenic effect of the parasite itself [14, 16]. As CanL has highly variable and frequently nonspecific clinical presentations, seasonal variation in antibody titers must be carefully considered by the clinician to avoid misdiagnosis of CanL active form or relapse, and consequently, unnecessary antileishmanial treatment [17].

The present study aimed to evaluate the influence of vector seasonality on laboratory findings related to CanL in seropositive dogs living in endemic areas.

## Methods

All dogs included in this study were from a rescue shelter in southern Italy (40° 0′ 45ʺ North, 18° 9′ 38ʺ East, Casarano, Apulia region). This area is endemic for CanL [18] and is a suitable environment for different *Phlebotomus* spp. (i.e., *Phlebotomus perniciosus*, *Phlebotomus perfiliewi*, and *Phlebotomus neglectus*), which transmit *L. infantum* [19]. In this geographical area, the sand fly season lasts from late May to late October, with two density peaks during July and August [19].

In September 2021, during the transmission season (T1), shelter dogs were physically examined, a clinical sign-based score for CanL, ranging from 0 (absence of clinical signs) to 19 (severely sick), was assigned [20], and a blood sampling was performed for laboratory tests to complete the dog’s health status.

Briefly, blood samples were collected from either the cephalic or jugular veins and placed in a K3 ethylenediaminetetraacetic acid (EDTA) tube (2 ml) to undergo routine hematology (i.e., complete blood count [CBC] with reticulocyte count), ESR, and modified Knott’s test. Exactly 5 ml of blood were placed in a plain tube to obtain serum after centrifugation (15 min at 1500 × *g*) to perform a complete biochemical panel, including acute-phase proteins (i.e., CRP and ferritin), serum capillary electrophoresis, and serology.

The erythrocyte sedimentation rate was measured using a point-of-care device (MINIPET, DIESSE, Diagnostica Senese S.p.A., Siena, Italy), and the reference interval was established as 0–10 mm/h [21]. Results from CBC (Siemens, ADVIA 2120), serum biochemical analysis (Beckman Coulter, Clinical Chemistry Analyzer AU680), and electrophoresis (SEBIA, Capillarys 2 Flex Piercing) were achieved with the same methods in all tested samples.

Serum samples were analyzed for *L. infantum* antibodies by IFAT as previously described [22]. They were considered positive if clear cytoplasmic and membrane fluorescence of *L. infantum* promastigotes from a cut-off dilution of 1:80 was evident. Positive sera were titrated by serial dilutions (i.e., 1:80, 1:160, 1:320, 1:640, 1:1280) up to 1:2560. Samples were considered negative if they failed to produce a positive result at a 1:80 dilution. All serological tests were read in a double-masked manner by two different operators.

Serum samples were also tested for *Anaplasma phagocytophilum* (MegaCor Diagnostik, Horbranz, Austria), and *Ehrlichia canis* (Biopronix Agrolabo, Scarmagno, Italy) antibodies by IFAT, and for the snail-borne pathogen *Angiostrongylus vasorum* diagnosed by the ELISA technique [23, 24].

Moreover, blood samples were processed using a modified Knott’s test for the detection and identification of circulating microfilariae (mfs) of *Dirofilaria* spp. as previously described [25]. Then, dogs without microfilariae were tested for *Dirofilaria immitis* antigen by ELISA (Filarcheck, Agrolabo, Scarmagno, Italy).

According to the laboratory results, dogs were considered finally eligible for this study at T1 if they were: (1) seropositive to *L. infantum*; (2) negative to *E. canis*, *A. phagocytophilum*, *Dirofilaria* spp., and *A. vasorum*; (3) not treated with antileishmanial drugs in the previous 6 months; and (4) not treated with insecticides or repellents in the previous 6 months.

In January 2022 (nontransmission season; T2), all dogs included underwent clinical examination, laboratory analyses, and serological testing as described above. During the study period, enrolled dogs did not receive any antileishmanial drug, as well as insecticides or repellents. Treatment decisions were indeed under the responsibility of the shelter’s attending veterinarian, in accordance with management policies and available financial resources. The researchers’ role was limited to clinical monitoring using the previously detailed diagnostic tools.

The normality of the results was checked using the Kolmogorov–Smirnov test. Laboratory parameters (i.e., albumin, albumin/globulin ratio, CRP, ESR, ferritin, gamma globulins, globulins, hematocrit, total iron, and total proteins) were normally distributed and reported as mean and standard deviation (M ± SD), and as frequencies and percentages (%) for categorical variables. The Wilcoxon matched-pairs signed-rank test was used to compare the differences between pairs of observations in the groups at time points (T1 and T2) for continuous variables. When testing the null hypothesis of no association, the probability level of error at two tails was 0.05. All the statistical computations were made using StataCorp. 2021. Stata Statistical Software: Release 17. College Station, TX, USA: StataCorp LLC.

## Results

**Fig. 1:**
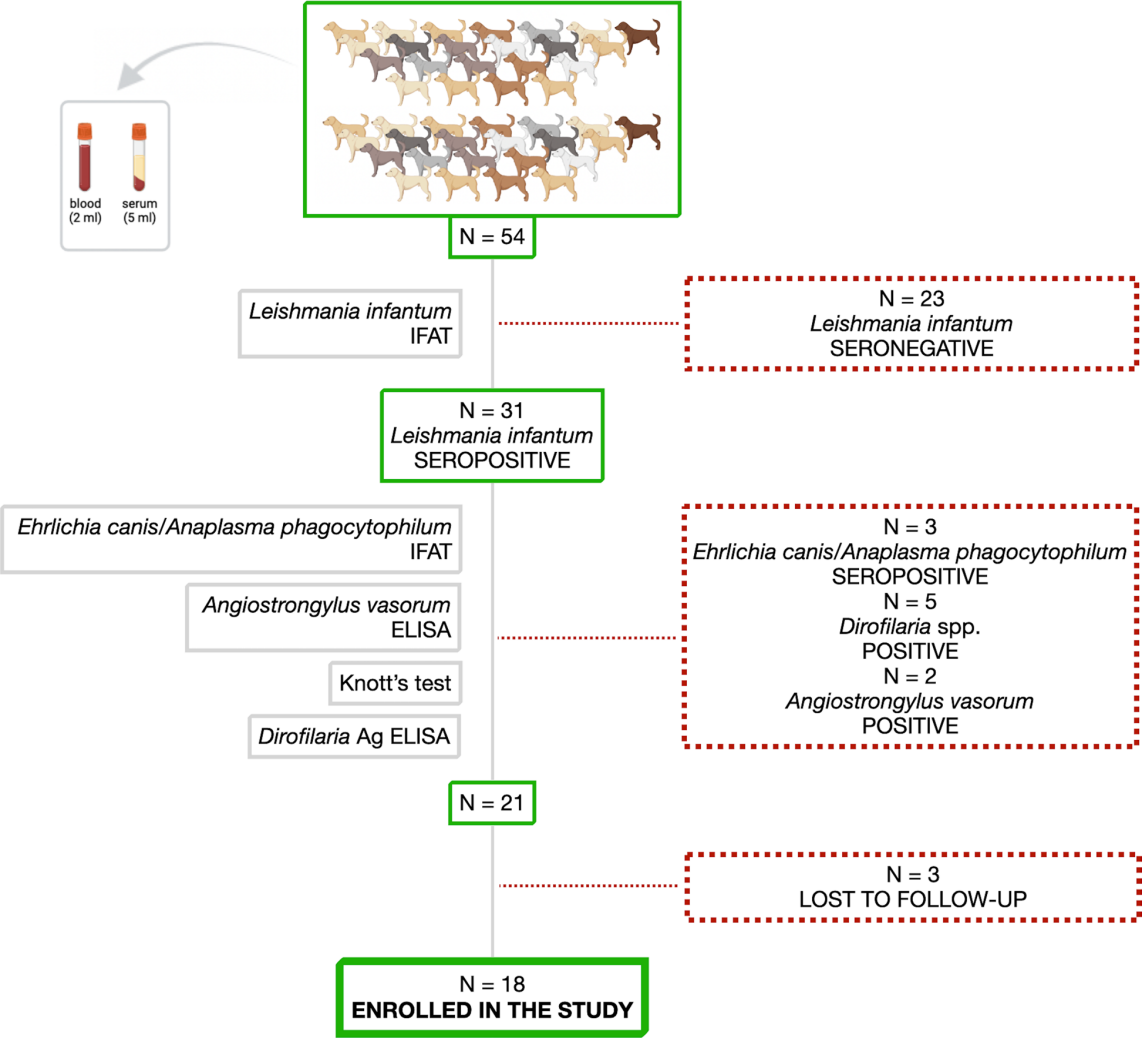
Flow chart showing the process for the inclusion of dogs in this study. Created in: https://biorender.com/

The enrollment procedure diagram is shown in Fig. 1. Of the 54 shelter dogs initially screened, 18 mixed-breed dogs were selected, thus enrolled in the study at T1, including 10 spayed females and 8 males (7 neutered and 1 entire) aged from 2 to 17 years (7.76 ± 3.99).

Variations in antibody titers, clinical score, and the main laboratory alterations related to CanL between T1 and T2 are shown in Tables 1 and 2. Gamma globulin percentage, ferritin, and ESR significantly reduced from T1 to T2, while albumin percentage and total iron significantly increased (Table 2). Although not statistically significant, total protein and CRP levels showed a slight decrease from T1 to T2, while the albumin/globulin ratio slightly increased (Table 2).

**Table 1:** Variation in antibody titers (Abs) against *Leishmania infantum* detected by indirect fluorescent antibody test (IFAT) according to sampling time and decrescent serial dilution (1:2560–1:80), clinical score (0–19), albumin percentage, gamma (γ)-globulins percentage, C-reactive protein (CRP), ferritin, and erythrocyte sedimentation rate (ESR)

N	Anti-*L. infantum* Abs by IFAT			Clinical score		Albumin (%)		γ-Globulins (%)		CRP (mg/dL)		Ferritin (ng/mL)		ESR (mm/h)	
	T1	T2	Abs T1 versus T0	T1	T2	T1	T2	T1	T2	T1	T2	T1	T2	T1	T2
#1	1/2560	1/80	Reduced	0	1	45.8	52.2	16.7	13.7	0.01	0.02	154	137	12	7
#2	1/1280	1/640	Reduced	3	1	28.7	32.9	28	22.3	4.3	2.86	143	105	5	10
#3	1/1280	1/160	Reduced	0	1	45.4	50.7	16.6	14.9	0.05	0.02	226	157	12	12
#4	1/1280	1/1280	Stable	1	2	50.7	53.9	17.9	14.4	0.05	0.02	231	107	11	5
#5	1/640	1/320	Reduced	1	1	43.3	46.8	20.7	20.9	0.02	0.16	358	198	11	9
#6	1/640	1/160	Reduced	0	1	45.5	50.8	24.1	17.6	0.01	0.08	141	126	10	3
#7	1/640	1/640	Stable	3	1	40.6	33.9	28.5	32.5	0.05	0.01	569	594	51	18
#8	1/640	1/320	Reduced	0	2	39.9	46.8	18.2	17	0.62	0.34	311	219	11	10
#9	1/640	1/640	Stable	0	1	44.8	51.3	15.7	14.4	0.03	0.02	147	153	11	14
#10	1/640	1/640	Stable	5	4	32.2	43.8	29.3	22.9	0.42	0.03	263	184	43	15
#11	1/320	1/320	Stable	0	0	44.5	46.9	12.8	9.5	0.51	0.28	NA	NA	NA	NA
#12	1/160	1/80	Reduced	1	2	46.1	47.2	15.7	15.3	0.03	0.32	198	144	14	1
#13	1/160	Neg	Reduced	1	0	48.8	49.9	17.3	16.3	0.44	0.09	167	94	11	11
#14	1/160	1/80	Reduced	1	1	41.4	48.8	17.3	13.7	0.04	0.23	378	204	10	9
#15	1/80	Neg	Reduced	0	2	58.9	52.1	10.2	12.4	0.04	0.18	156	101	10	9
#16	1/80	1/80	Stable	1	1	50.6	52.3	15.8	14.2	0.03	0.02	156	143	12	12
#17	1/80	1/80	Stable	0	0	45.7	44.7	12.9	13.4	0.03	0.23	NA	NA	NA	NA
#18	1/80	1/80	Stable	0	0	55.8	55.0	11.3	11.2	0.23	0.06	NA	NA	NA	NA

Enrollment of seropositive dogs was in the transmission season (T1), and follow-up was in the nontransmission season (T2)

*NA* Not available

**Table 2:** Variation in the main laboratory findings related to canine leishmaniosis between the transmission season (September 2021, T1) and nontransmission season (January 2022, T2)

Parameters^a^	Reference values	Time points			
		T1	T2	*P* ^^^	*Z*
Total proteins (g/dL)	5.7–7.3	6.80 ± 0.48	6.63 ± 0.32	0.14	1.86
Albumin (g/dL)	2.8–3.7	2.88 ± 0.39	2.90 ± 0.38	0.58	-0.62
Globulins (g/dL)	2.8–3.9	3.92 ± 0.68	3.73 ± 0.50	0.24	1.72
A/G	0.70–1.30	0.76 ± 0.20	0.80 ± 0.17	0.21	-1.37
Albumin (%)	49.0–60.0	44.93 ± 7.20	47.78 ± 6.07	0.03^*^	-2.33
Gamma globulins (%)	6.4–14.5	18.28 ± 5.74	16.48 ± 5.36	0.03^*^	2.37
CRP (mg/dL)	0.01–0.45	0.38 ± 1.00	0.28 ± 0.65	0.48	0.89
Ferritin (ng/mL)	80–270	239.87 ± 120.57	177.73 ± 121.61	0.007^*^	3.07
Total iron (mcg/dL)	90–230	169.78 ± 36.93	185.94 ± 41.60	0.007^*^	-1.94
HCT (%)	38–54	48.56 ± 6.38	48.25 ± 3.97	0.99	0.02
ESR (mm/h)	0–10	15.60 ± 12.98	9.67 ± 4.47	0.04^*^	2.16

^*^Statistically significant

^a^ As mean and standard deviation (M ± SD)

*A/G* albumin/globulins ratio, *CRP* C-reactive protein, *HCT* hematocrit, *ESR* erythrocyte sedimentation rate

^^^ Wilcoxon matched-pairs signed-rank test

The anti-*L. infantum* antibody titer of the 18 dogs was determined at both sampling times (Table 1) and decreased in 10 individuals (55.5%), with 2 becoming seronegative (20%), and remained stable in 8 dogs (44.4%).

During the trial, dogs maintained a good state of health with a mean clinical score of 1.1 and 1.2 at T1 and T2, respectively (Table 1). A total of 8 out of 18 dogs experienced a slight increase in their clinical score, while 6 and 4 dogs showed the same or a reduced clinical score, respectively (Table 1).

## Discussion

This study reported significant variations in some clinicopathological abnormalities in dogs with CanL, during and after sand fly transmission. These results overlap seasonal variations in anti-*L. infantum* antibody titers, previously described in the same area [14, 15].

Laboratory alterations are relevant for veterinary practitioners in the diagnosis and staging of CanL [4–6], mainly in areas where multiple canine vector-borne disease-causing pathogens co-occur [26]. Though clinical presentation and type of laboratory findings are quite variable in CanL, a group of routine hematology (e.g., normocytic normochromic poorly or nonregenerative anemia) and/or clinical chemistry (e.g., hyperproteinemia, hyperglobulinemia, hypoalbuminemia, decreased A/G ratio, renal azotemia, elevated liver enzyme activities) alterations are considered highly indicative of active leishmaniosis, hence representing an alarm bell for antileishmanial treatments in *L. infantum* seropositive dogs, with or without clinical signs [2, 6, 27].

Dogs examined here, yet untreated, maintained a good state of health and experienced a change in several hematobiochemical markers with a statistically significant improvement of gamma globulin, albumin, total iron, ferritin, ESR, and CRP in the nontransmission season (T2). Furthermore, although not statistically significant, a similar decreasing trend was also present for total proteins and albumin/globulin ratio, with more than half of the dogs enrolled showing a reduction in anti-*L. infantum* antibody titers at T2. The above could be owing to an increase in the parasite load in dogs exposed to infected sand fly bites, potentially leading to a “transient” onset of common laboratory abnormalities. In such cases, the development of an effective immune response may eventually resolve these transient changes during the nontransmission season. This hypothesis could be supported by the fact that dogs enrolled in the present study did not present the plethora of signs indicative of clinically evident disease (i.e., general, cutaneous and mucocutaneous, ocular findings) [2, 6] during the entire observation period, as suggested by very low clinical score values (Table 1).

Overall, the available data suggest that anti-*L. infantum* antibody titers are affected by exposure to sand fly saliva during the transmission season [16], although the influence on clinical outcome in infected dogs is poorly understood. Under laboratory conditions, skin lesions caused by *Leishmania major* developed more rapidly and reached a larger size in BALB/c mice exposed to repeated sand fly bites compared with unexposed ones [28].

Seasonal variation in antibody titers and laboratory abnormalities has potential practical implications, including misdiagnosis and therapeutic mismanagement of dogs with CanL. According to the main available guidelines, dogs with high *Leishmania* antibody titers (i.e., four-fold higher than the cut-off threshold), or with clinicopathological alterations suggestive of leishmaniosis should be considered “sick” in the need of antileishmanial treatment, even in the absence of clinical signs (e.g., generalized lymphadenomegaly, exfoliative dermatitis, weight loss, onychogryphosis, alopecia, ulcers, and ocular alterations) and without the confirmation of the presence of the parasite by direct tests [2, 6]. According to the above, 4 out of the 18 dogs enrolled at T1 (#1–#4) should have been classified as “sick” and treated for leishmaniosis, as they had an antibody titer of ≥ 1:1280. However, considering the seasonal variation in antibody titers [14, 15], the anti-*L. infantum* antibody titers recorded in T1 (during the sand fly season) in these four dogs could have been influenced by seasonality. Moreover, when reassessed at T2 (outside the sand fly season), three of the above cases (#1, #3, #4) showed a reduction in antibody titers and/or an improvement in laboratory parameters (Table 1), suggesting that they may not have required any treatment.

Therefore, sampling time should be carefully assessed in the CanL clinical diagnosis and staging processes to avoid unnecessary antileishmanial treatment, which could predispose to adverse effects and drug resistance. In addition, the seasonal variation should also be considered in field studies evaluating the efficacy of preventive and/or treatment measures for CanL, as it may influence the interpretation of product effectiveness. However, though this preliminary study provides useful insights, it involved a limited number of dogs from a single shelter. Further investigations on larger and more diverse populations are warranted to confirm and extend these findings. Incorporating vector data (e.g., presence and density) and additional time points could also be valuable.

## Conclusions

Anti-*L. infantum* antibody titers and clinicopathological alterations in seropositive dogs living in endemic areas for leishmaniosis may vary. This variation may be related to vector seasonality and, consequently, dogs’ exposure to sand fly saliva and potential reinfections. These results reinforce the importance of considering the sampling season in the clinical evaluation and management of dogs affected by leishmaniosis to avoid misdiagnosis and unnecessary antileishmanial treatments.

## Data Availability

Data supporting the main conclusion of this study are available in the manuscript.

## Abbreviations

APP: Acute phase proteins
CanL: Canine leishmaniosis
CBC: Complete blood count
CRP: C-reactive protein
ELISA: Enzyme-linked immunosorbent assay
ESR: Erythrocyte sedimentation rate
IFAT: Indirect immunofluorescence antibody test
MFS: Microfilariae
